# Naringenin Alleviates Renal Ischemia Reperfusion Injury by Suppressing ER Stress-Induced Pyroptosis and Apoptosis through Activating Nrf2/HO-1 Signaling Pathway

**DOI:** 10.1155/2022/5992436

**Published:** 2022-10-10

**Authors:** Banghua Zhang, Shanshan Wan, Hao Liu, Qiangmin Qiu, Hui Chen, Zhiyuan Chen, Lei Wang, Xiuheng Liu

**Affiliations:** ^1^Department of Urology, Renmin Hospital of Wuhan University, Wuhan 430060, China; ^2^Hubei Key Laboratory of Digestive System Disease, Wuhan 430060, China; ^3^Department of Ophthalmology, Renmin Hospital of Wuhan University, Wuhan 430060, China; ^4^Wuhan University Institute of Urological Disease, Wuhan 430060, China

## Abstract

Endoplasmic reticulum (ER) stress, pyroptosis, and apoptosis are critical molecular events in the occurrence and progress of renal ischemia reperfusion (I/R) injury. Naringenin (4′,5,7-trihydroxyflavanone) is one of the most widely consumed flavonoids with powerful antioxidant and anti-inflammatory activities. However, whether naringenin is able to relieve renal I/R injury and corresponding mechanisms have not been fully clarified. This study was aimed at exploring its role and relevant mechanisms in renal I/R injury. The C57Bl/6 mice were randomly assigned to receive administration with naringenin (50 mg/kg/d) or sterile saline (1.0 mL/d) for 3 d by gavage and suffered from renal I/R surgery. One specific ER stress inhibitor, 4-phenylbutyric acid (4-PBA, 100 mg/kg/d), was intraperitoneally administered to validate the regulation of ER stress on pyroptosis and apoptosis. Cultured HK-2 cells went through the process of hypoxia/reoxygenation (H/R) to perform cellular experiments with the incubation of naringenin (200 *μ*M), 4-PBA (5 mM), or brusatol (400 nM). The animal results verified that naringenin obviously relieved renal I/R injury, while it refined renal function and attenuated tissue structural damage. Furthermore, naringenin treatment inhibited I/R-induced ER stress as well as pyroptosis and apoptosis as indicated by decreased levels of specific biomarkers such as GRP78, CHOP, caspase-12, NLRP3, ASC, caspase-11, caspase-4, caspase-1, IL-1*β*, GSDMD-N, BAX, and cleaved caspase-3 in animals and HK-2 cells. Besides, the upregulated expression of Nrf2 and HO-1 proteins after naringenin treatment suggested that naringenin activated the Nrf2/HO-1 signaling pathway, which was again authenticated by the usage of brusatol (Bru), one unique inhibitor of the Nrf2 pathway. Importantly, the application of 4-PBA showed that renal I/R-generated pyroptosis and apoptosis were able to be regulated by ER stress in vivo and in vitro. In conclusion, naringenin suppressed ER stress by activating Nrf2/HO-1 signaling pathway and further alleviated pyroptosis and apoptosis to protect renal against I/R injury.

## 1. Introduction

Acute kidney injury emerges as a fairly intractable and worrisome clinical problem, which is often accompanied by varieties of syndromes such as urinary tract obstruction, cardiorenal syndrome, and sepsis. AKI occurs in more than 50% of patients in intensive care and in 10-15% of patients [1]. Blood flow to the kidney is suddenly reduced or stopped in renal I/R injury. After blood supply is restored, the injury is further aggravated and can develop into acute kidney injury and other serious kidney diseases [2]. Although the pathogenesis of renal I/R injury remains to be clearly elucidated, relevant studies have confirmed that oxidative stress, ER stress, mitochondrial dysfunction, ion accumulation, pyroptosis, and apoptosis are critical molecular mechanisms. Furthermore, relevant articles have authenticated that ER stress proves to be the critical regulatory mediator in renal I/R injury as evidenced by reduced renal I/R injury after effectively inhibiting ER stress [3–5]. In view of the significant mortality and morbidity of related diseases including AKI caused by renal I/R injury, it is increasingly necessary to completely understand its cellular pathophysiological mechanisms and persistently explore new therapeutic strategies of renal I/R injury [6, 7].

ER stress, pyroptosis, and apoptosis are substantial and interrelated processes in the occurrence and etiopathogenesis of renal I/R injury [3, 8]. When the internal environment has changes including disruption of calcium homeostasis, hypoxia, and oxidative stress, endoplasmic reticulum homeostasis is imbalanced, leading to an abnormal increase in intracellular misfolded or unfolded proteins, which ultimately induce ER stress [9]. When cells are continuously under pathological stress, apoptotic pathways are triggered, resulting in massive accumulation of unfolded proteins and apoptosis [10]. In addition, ER stress has been verified to have participation in the activation of NF-*κ*B signaling pathway and NLRP3 inflammasome, which in turn cause pyroptosis [11, 12].

As one of the largely consumed flavonoids which widely exist in Citrus genus, naringenin possesses a broad prospect of clinical application owing to its excellent bioavailability, low cytotoxicity, and remarkable anti-inflammatory and antioxidant properties [13–15]. Recent researches in different fields have testified that naringenin has showed noteworthy protective effects in cardiovascular diseases, neurological disorders, diabetes mellitus, virus infection, and I/R injury. These functions are mainly achieved through mitigating inflammatory response, oxidative stress, ER stress, and apoptosis [16–18]. Meanwhile, naringenin is able to relieve myocardial I/R injury by inhibiting oxidative stress, ferroptosis, and ER stress via AMPK-SIRT3, Nrf2/GPX4, and PI3K/AKT pathways [19–21]. Pretreatment of naringenin protects retinal and brain against I/R injury as well as ameliorating ischemic stroke [22, 23]. Nevertheless, whether it can alleviate renal I/R injury and the corresponding mechanisms demands to be further authenticated.

Therefore, we innovatively investigated the protective influence and the underlying mechanisms of naringenin treatment on renal I/R-generated ER stress, pyroptosis, and apoptosis by establishing the typical renal I/R model in C57Bl/6 mice and an H/R model in HK-2 cells, so as to provide some new perspectives into the pathogenesis occurrence of renal I/R injury and a theoretical basis for naringenin in the clinical treatment of kidney ischemic diseases.

## 2. Materials and Methods

### 2.1. Antibodies and Reagents

Naringenin (HPLC ≥ 98%) used in this study was acquired from Solarbio (IN0350, Solarbio Life Sciences, Beijing). Annexin V-FITC/PI apoptosis kit that was applied in flow cytometry was supplied by Multi Sciences Biotech (Hangzhou, China). The 5x protein loading buffer, BCA protein quantification kit, radio-immunoprecipitation assay (RIPA) buffer, SDS-PAGE gel preparation kit, 10x TBS with Tween-20 buffer, and phenylmethanesulfonyl fluoride (PMSF) all were bought from Servicebio Technology (Wuhan, China). Fetal bovine serum was purchased from Invitrogen (MA, USA). Polyvinylidene difluoride (PVDF) membrane and chemiluminescent HRP substrate were obtained from Millipore (Billerica, MA, USA). Sources and usage of other reagents are described in detail in the following instructions.

### 2.2. Renal I/R Injury Mice Model

Eight-week-old adult male C57Bl/6 mice (22-24 g) were provided by the First Clinical College Experimental Animal Center of Wuhan University. The project was approved by Bioethics Committee of the Renmin Hospital of Wuhan University. These mice were provided with adequate food, sufficient water, appropriate room temperature (22-23°C), and light for a fixed period of time (the time is all 12 h of darkness or light for one cycle) according to the Laboratory Animal Guidelines.

The renal I/R model was built as we did in the previous studies; we selected 30 min of ischemia followed by suturing the incision and one full day (24 h) of reperfusion because renal I/R damage of this model was obvious [24, 25]. 0.2% pentobarbital sodium (60 mg/kg) was selected to anesthetize the mice in the experiments by intraperitoneal injection followed by placing the experimental mice on a thermostatic blanket. After skin preparation, a longitudinal abdominal incision was made to expose and separate bilateral kidneys and renal arteries. Only the right kidney was removed without subsequent treatment, and the mice were followed by the suture of abdominal incision in the Sham group. The left renal artery was clamped with a noninvasive vascular clip after right-sided nephrectomy in all I/R groups, and the kidney rapidly turned into black and purple after clamping. The arterial clamp was removed to restore blood supply after 30 min of ischemia, and the kidney quickly turned red. After the incision was sutured, 0.5 mL normal saline was injected intraperitoneally for liquid resuscitation. Blood and kidney tissue were collected immediately after 24 h of reperfusion.

### 2.3. Mice Treatment

All C57Bl/6 mice were randomly divided into 5 groups, which consisted of the Sham group, I/R group, I/R+NS (sterile saline) group, I/R+NRG (naringenin) group, and I/R+4-PBA (4-phenylbutyric acid) group, n=5. The Sham group received the above-mentioned treatment after normal feeding. Vehicle (1.0 mL/d, sterile saline) or naringenin (50 mg/kg/d, dilution in NS) was conducted by oral gavage for 3 d in the I/R+NS group or I/R+NRG group. Mice were injected intraperitoneally with 4-PBA (100 mg/kg, dilution in phosphate-buffered saline) 24 h before undergoing I/R surgery in the I/R+4-PBA group. The dose of 4-PBA was chosen for this study because there were previous articles about 4-PBA treatment in adult animals [26].

### 2.4. Assessment of Renal Function

Fresh blood from all experimental mice was collected immediately after 24 h reperfusion, followed by 20 min of centrifugation at 3000 r/min. The corresponding kits were purchased from Jiancheng Bioengineering Institute in Nanjing of China to measure the creatinine (Cr) and blood urea nitrogen (BUN) levels according to the product instructions.

### 2.5. HE Staining

After embedding the formaldehyde-fixed mouse kidney tissues with paraffin, we prepared 4 *μ*m thick sections and employed hematoxylin-eosin (H&E) to stain them. The pathological changes of renal tissue were observed under microscope. Two professional renal pathologists randomly selected 8 fields from each section to observe the pathological changes of renal tubule-interstitial lesions and performed semiquantitative scores of renal tubule-interstitial lesions.

### 2.6. Cell Culture and In Vitro H/R Model

As a cell line commonly used to construct in vitro model of renal I/R injury [27, 28], HK-2 cells utilized in H/R model were supplied by China Center for Type Culture Collection (CTCC, China). HK-2 cell line is also known as human renal proximal tubular epithelial cell line. DMEM medium called as Dulbecco's modified Eagle's medium was chosen to culture HK-2 cells, which need to undergo 12 h of incubation in serum-free medium in a three-gas incubator (5% CO_2_, 94% N_2_, 1% O_2_) to complete this step of hypoxia. Reoxygenation was accomplished by changing the serum-free medium into normal medium containing 10% fetal bovine serum (FBS) immediately after completion of hypoxia, and then, HK-2 cells in the H/R groups were cultured normally in an ordinary incubator with 5% CO_2_, 74% N_2_, and 21% O_2_ at 37°C. Meanwhile, HK-2 cells in the control group of cellular models were cultured in the normal environment using complete medium with 10% FBS at all times. The 12 h of hypoxia and 4 h of reoxygenation were chosen because our previous studies showed that both apoptosis and pyroptosis were relatively pronounced in such a model [25, 29]. Different concentrations of NRG or 4-PBA (5 mM, dilution in DMSO) were added to the medium 24 h before model construction [30], and then, they were incubated with or without brusatol (400 nM), 2 h before hypoxia [25].

### 2.7. Quantitative Real-Time PCR Analysis

RNA extraction kit (G3013, Servicebio, Wuhan) was purchased to extract the total RNA in relevant groups of animals and HK-2 cells according to the standard procedures. 1,000 ng of extracted RNA was then reversely transcribed into cDNA by the application of one SweScript RT II First Strand cDNA Synthesis Kit (G3333-100, Servicebio, Wuhan). Primers for human and mouse genes were designed and synthesized by Sangon Biotech (Shanghai, China). 20 *μ*L qPCR reaction system including 2x Universal Blue SYBR Green qPCR Master Mix (G3326-05, Servicebio, Wuhan) was performed to detect the relative mRNA levels of target genes by qPCR Detection System (Bio-Rad, USA). 2^-△△CT^ method was selected to quantify the levels of gene mRNA expression relative to GAPDH. The sequences of primers used in our study are shown in Tables 1 and 2.

### 2.8. Western Blot Analysis

The kidney tissues that were preserved in liquid nitrogen at -80°C were cut and homogenized, followed by the addition of precooled RIPA buffer containing PMSF and centrifugation at 6,000 r/min for 20 min. The treated cells in all groups were collected for protein extraction following a standard procedure. After the protein content was determined through the utilization of the BCA kit, 30 *μ*g of the unmeasured protein was mixed with the loading buffer 5x and then boiled in a water bath at 100°C for 10 min. The proteins were then separated using 10% sodium dodecyl sulfate-polyacrylamide gels (SDS-PAGE) and transferred electrophoretically to PVDF membranes. In order to eliminate nonspecific binding of the target proteins with the primary antibodies, Protein Free Rapid Blocking Buffer (1x) (PS108P, Epizyme Biomedical Technology, Shanghai) was used to block for 30 min at 37°C. Membranes were infiltrated with a specific dilution of primary antibodies overnight at 4°C. The dilutions and sources of all antibodies are as follows: GAPDH (10494-1-AP, 1 : 8000, Proteintech Group), GRP78 (11587-1-AP, 1 : 2000, Proteintech Group), CHOP (15204-1-AP, 1 : 2000, Proteintech Group), BAX (50599-2-Ig, 1 : 5000, Proteintech Group), cleaved caspase-3 (WL02117, 1 : 500, Wanleibio), HO-1 (10701-1-AP, 1 : 3000, Proteintech Group), Bcl-2 (26593-1-AP, 1 : 2000, Proteintech Group), Nrf2 (16396-1-AP, 1 : 5000, Proteintech Group), caspase-4 (sc-56056, 1 : 200, Santa Cruz), NLRP3 (#15101, 1 : 1000, Cell Signaling Technology), caspase-11 (sc-56038, 1 : 400, Santa Cruz), cleaved caspase-1 (sc-56036, 1 : 400, Santa Cruz), caspase-12 (sc-21747, 1 : 400, Santa Cruz), mature IL-1*β* (#12242, 1 : 1000, Cell Signaling Technology), ASC (sc-514414, 1 : 200, Santa Cruz), GSDMD-N (#39754, 1 : 1000, Cell Signaling Technology), and KIM-1 (AF1817, MAB1750, 1 : 1000, R&D Systems). The PVDF membranes were then placed into the diluted goat anti-rabbit or goat anti-mouse secondary antibody (SA00001-2, SA00001-1, 1 : 2000, Proteintech Group) and incubated for 1 h at 37°C. All membranes were flushed with TBST 1x buffer three times for 10 min each to lessen nonspecific binding. Chemiluminescent HRP substrate was applied to visualize all blots. Protein levels were analyzed and quantified by Image Lab Software (NIH, USA).

### 2.9. Detection of Caspase-1 Activity

The caspase-1 activity in various treatment groups was able to be detected by the caspase-1 activity assay kit (C1102, Beyotime, Shanghai) according to the attached detailed instructions. Briefly, after we collected the treated kidney tissue and HK-2 cells, the 100 *μ*L reaction system was configured after successively adding the reagents from this kit according to the manufacturer's instructions. The samples were incubated at 37°C for 90 min. The absorbance at the wavelength of 405 nm was determined to assess the levels when the color change is obvious.

### 2.10. Cell Viability Assay

Cell viability in different groups of cellular experiments was measured by one CCK-8 cell viability assay kit, which was purchased from Nanjing Jiancheng Bioengineering Institute (Nanjing, China). The cell suspension of 10,000 cells was added to every well of the 96-well plates and cultured for 48 hours, followed by the various concentrations of NAR and relevant treatments. After being added with 10 *μ*L of CCK-8 reagent per well, the cells were continued to be incubated in the dark for 4 hours. The absorbance of the treated cells in 96-well plates at 450 nm was quantified by the PerkinElmer Microplate reader (PerkinElmer Victor 1420, USA) to determine the cell viability.

### 2.11. Measurement of Caspase-3 Activity

One caspase-3 activity assay kit (C1116, Beyotime, Shanghai) was bought to assess the caspase-3 activity. According to the accompanying thorough instructions, tissue or cell samples treated with precooled RIPA buffer were centrifuged for 15 min at 12,000 r/min at 4° C, and the transferred supernatant was immediately used to configure the 100 *μ*L system containing reagents in this kit. The samples were incubated at 37°C for 120 min. The levels were quantified by the utilization of the absorbance at 405 nm.

### 2.12. Flow Cytometry

Flow cytometry was taken to measure the apoptosis degree of HK-2 cells in various intervention groups through the Annexin V-FITC/PI apoptosis kit by referring to the attached instructions. HK-2 cells with different pretreatments were washed three times in PBS 1x buffer. Then, 5 × 10^3^ cells including cells inside the culture supernatant were collected. After being resuspended by 1,000 *μ*L of 1x binding buffer, cells in each group were added with 20 *μ*L PI and 10 *μ*L Annexin V-FITC, followed by 15 min incubation at 37°C in the dark. The FACS flow cytometer (Bio-Rad, USA) was applied to detect the apoptotic cells.

### 2.13. Statistical Analysis

The experimental data in animal and cell experiments were quantified and processed by software GraphPad Prism version 8.0 (CA, USA). The results were presented as mean + standard deviation (SD). One-way analysis of variance (ANOVA) followed by Tukey's test was used to perform the statistical analysis. *P* < 0.05 indicated a statistically significant comparison between the different groups.

## 3. Results

### 3.1. Naringenin Ameliorated Renal Ischemia Reperfusion Injury in Mice

**Figure 1:**
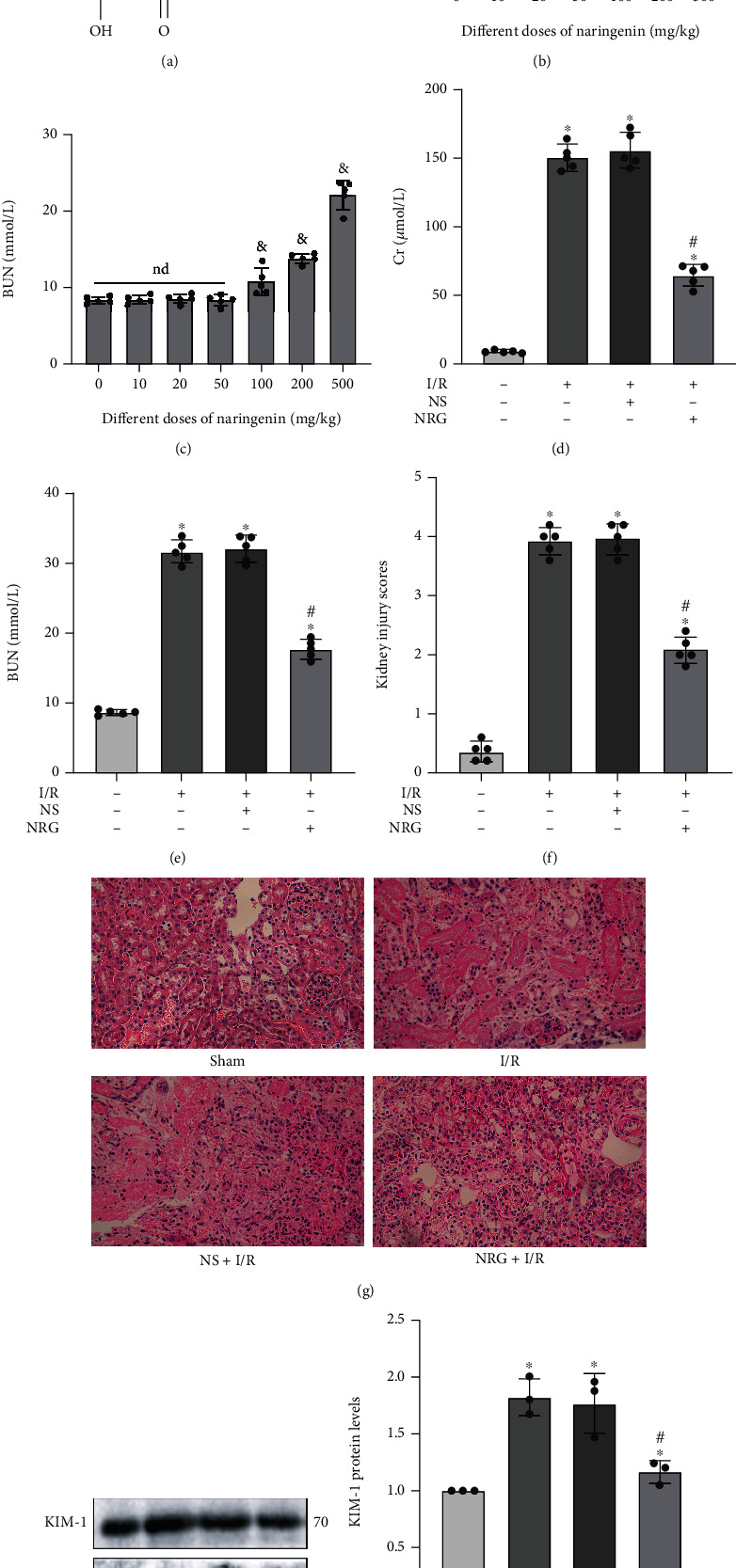
Naringenin ameliorated renal ischemia reperfusion injury in mice. (a) The structural formula of naringenin (NRG). (b, c) The influence about a variety of doses of naringenin on the levels of serum Cr as well as serum BUN in normally fed eight-week-old C57Bl/6 mice. (d, e) The levels of kidney biomarkers such as serum Cr and BUN declined notably in the NRG+I/R group. (f, g) H&E staining (×400) used in kidney histological staining showed that kidney tissue damage was mitigated in the NRG+I/R group. (h) Western blot analysis utilized in protein detection validated that the protein levels of KIM-1 decreased significantly in the NRG+I/R group. Values measured during animal experiments were carried out as mean ± SD, *n* = 3 − 5. ^&^*P* < 0.05, compared with 0 mg/kg group; ^∗^*P* < 0.05, compared with the Sham group; ^#^*P* < 0.05, relative to the NS+I/R group; nd: no statistical difference.

The structural formula of naringenin as a flavonoid is as follows (Figure 1(a)). In order to determine the appropriate dose that could be administered in mice, we explored the concentration gradient to test effects of naringenin on renal function. The results of the pretest showed that naringenin did not significantly affect renal function at a dose of 50 mg/kg, as evidenced by Cr and BUN levels in normally fed mice (Figures 1(b) and 1(c)). Mice were then treated with NRG (50 mg/kg/d) or NS (1.0 mL/d) by gavage for 3 d as mentioned above, followed by construction of typical renal I/R model. Cr and BUN, the serum markers of kidney injury, were clearly increased in the renal I/R model, but their levels were obviously downregulated in the NRG+I/R group (Figures 1(d) and 1(e)). H&E staining authenticated that pretreatment with NRG effectively improved the renal tissue morphology, which exhibited loss of brush border and tubular dilatation in the kidneys exposed to I/R surgery (Figures 1(f) and 1(g)). Consistent with these results, there was also an evident reduction in the protein levels of kidney injury molecule 1 (KIM-1) after NRG administration (Figure 1(h)). In conclusion, these experimental results confirmed that NRG gavage treatment could availably alleviate the pathological damage and kidney dysfunction in mice after the construction of I/R model.

### 3.2. Naringenin Effectively Attenuated Renal I/R-Generated ER Stress and Activated Nrf2/HO-1 Signaling Pathway in Mice

**Figure 2:**
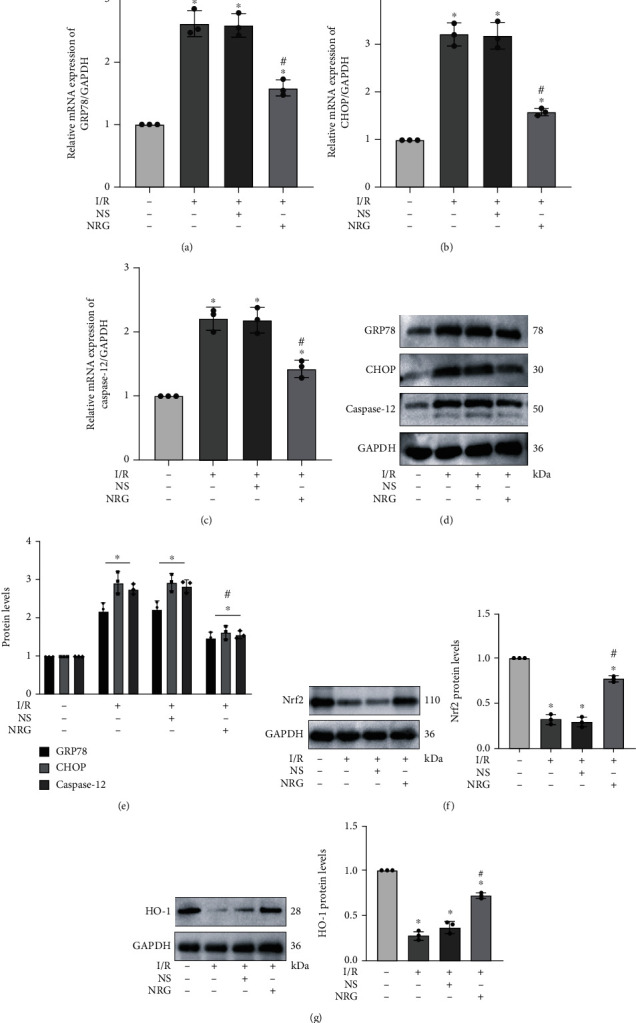
Naringenin effectively attenuated renal I/R-generated ER stress and activated Nrf2/HO-1 signaling pathway in mice. (a–c) qRT-PCR authenticated that pretreatment of naringenin effectively reduced the mRNA expression of GRP78, CHOP, and caspase-12 induced by renal I/R surgery. (d, e) Western blot analysis proved that naringenin administration downregulated ER stress-related proteins including GRP78, CHOP, and caspase-12 during renal I/R injury. (f, g) The protein levels of Nrf2 and HO-1 in renal tissues were displayed through western blot analysis in C57Bl/6 mice. Values measured during animal experiments were carried out as mean ± SD, *n* = 3 (three times measurements). ^∗^*P* < 0.05, compared with the Sham group; ^#^*P* < 0.05, relative to the NS+I/R group.

Subsequently, to identify corresponding mechanism by which NRG relieved renal I/R injury, members of this subject group performed qRT-PCR and western blot analysis to detect specific markers related to ER stress in renal I/R injury. Relevant studies revealed that ER stress plays the key role in renal I/R injury [3, 8], and NRG was testified by some studies to possess a meaningful role in regulating ER stress [31, 32]. By detecting the mRNA levels, we could clearly see that ER stress-specific markers including GRP78, CHOP, and caspase-12 were distinctly activated after I/R injury. Conversely, the results validated that NRG employment notably restrained the ER stress produced by I/R construction, as evidenced by the markedly lessened mRNA levels of both GRP78, CHOP, and caspase-12 (Figures 2(a)–2(c)). Western blot analysis further confirmed the effect of naringenin on inhibiting ER stress induced by renal I/R injury (Figures 2(d) and 2(e)). Moreover, we discovered that pretreatment of NRG reactivated restrained Nrf2/HO-1 signaling pathway in renal I/R injury (Figures 2(f) and 2(g)).

### 3.3. Naringenin Significantly Inhibited Pyroptosis and Apoptosis Induced by Renal I/R Injury in Mice

**Figure 3:**
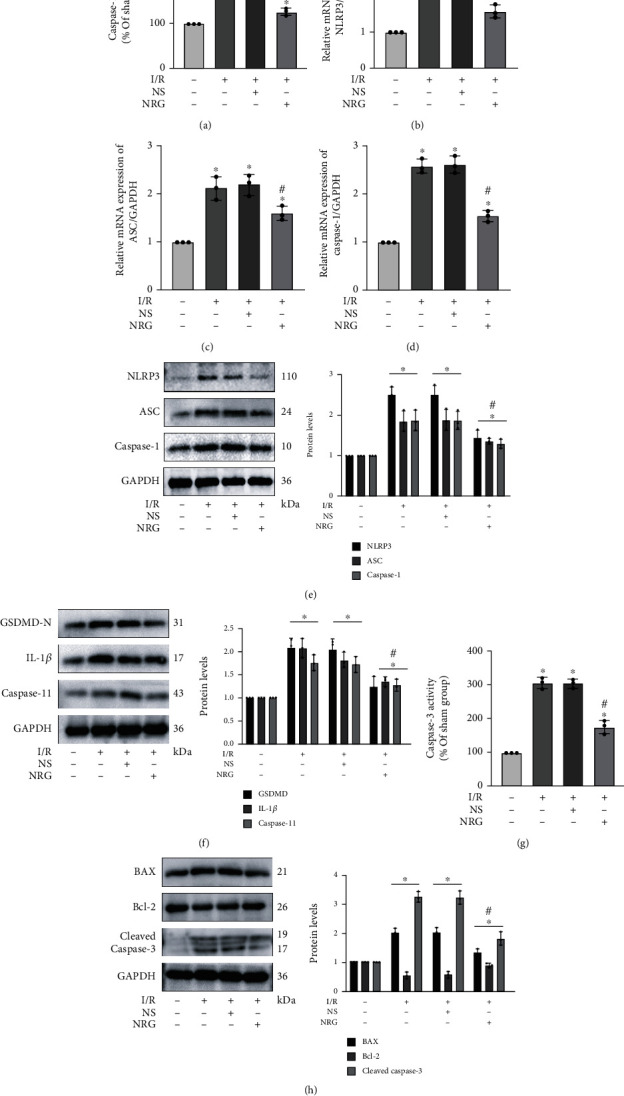
Naringenin significantly inhibited pyroptosis and apoptosis induced by renal I/R injury in mice. (a) The levels of caspase-1 activity in the I/R group exhibited obvious reduction after naringenin treatment. (b–d) Naringenin significantly inhibited the mRNA expression of pyroptosis-related markers such as NLRP3, ASC, and caspase-1 in renal I/R injury. (e, f) Renal I/R-generated pyroptosis was obviously ameliorated by naringenin application as evidenced by the decreased protein expression of NLRP3, ASC, caspase-1, GSDMD-N, caspase-11, and IL-1*β* in extracted kidney tissues. (g) The usage of naringenin remarkably restrained caspase-3 activity in renal I/R injury. (h) Western blot analysis utilized in protein detection of kidney tissue was selected to detect the protein levels of Bcl-2, BAX, and cleaved caspase-3 in the four groups. Values measured during animal experiments were carried out as mean ± SD, *n* = 3 (three times measurement). ^∗^*P* < 0.05, compared with the Sham group; ^#^*P* < 0.05, relative to the NS+I/R group.

Next, pyroptosis-associated and apoptotic markers were further detected. The results suggested that the levels of caspase-1 activity in the I/R group exhibited obvious reduction after naringenin treatment (Figure 3(a)). The results of qRT-PCR authenticated that naringenin significantly inhibited the mRNA expression of pyroptosis-related markers such as NLRP3, ASC, and caspase-1 in renal I/R injury (Figures 3(b)–3(d)). The function of NRG on alleviating pyroptosis was again confirmed by the decreased protein expression of NLRP3, ASC, caspase-1 p10, GSDMD-N, caspase-11, and mature IL-1*β* in the NRG+I/R group (Figures 3(e) and 3(f)). Meanwhile, the usage of NRG remarkably restrained caspase-3 activity in renal I/R injury (Figure 3(g)). NRG application also abrogated the enhanced protein levels of apoptotic protein cleaved caspase-3 induced by I/R exposure as well as BAX, while the decreased expression of Bcl-2 protein in the I/R group was restored in the NAR+I/R group (Figure 3(h)).

### 3.4. Renal I/R-Generated Pyroptosis and Apoptosis Could be Regulated by ER Stress in Mice

**Figure 4:**
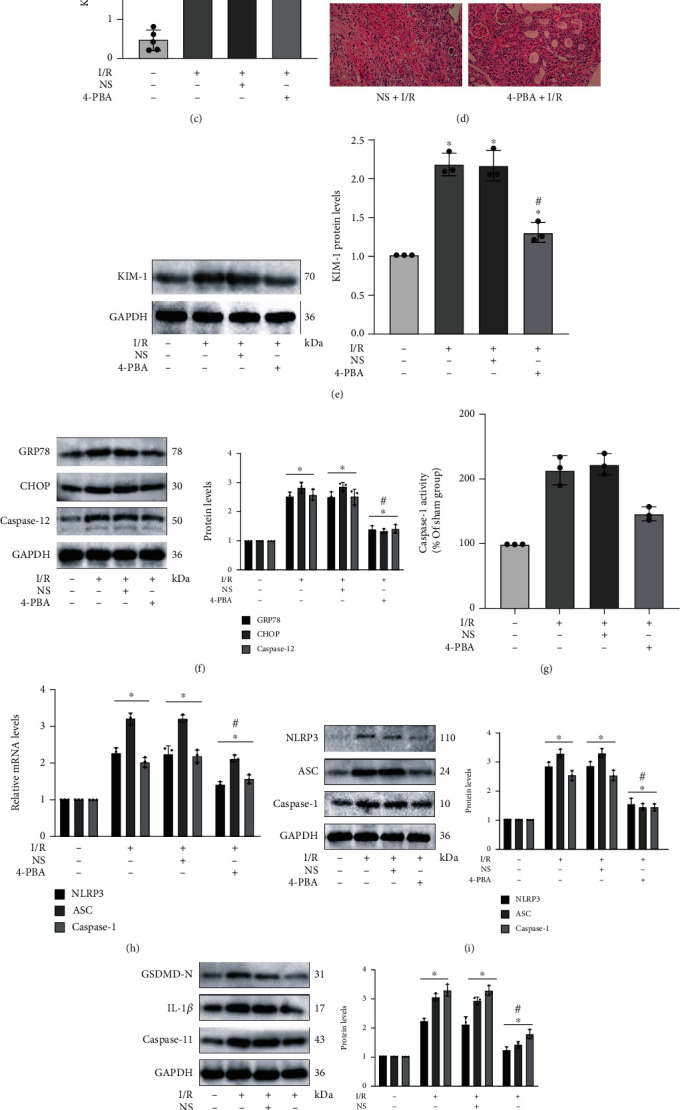
Renal I/R-generated pyroptosis and apoptosis could be regulated by ER stress in mice. Mice in the 4-PBA+I/R group were injected intraperitoneally with 4-PBA (100 mg/kg, dilution in phosphate-buffered saline) 24 h before undergoing renal I/R surgery. (a, b) The levels of serum Cr and serum BUN in renal I/R injury decreased notably after inhibiting ER stress by 4-PBA application. (c, d) Quantitative analysis of tubular injury scores and representative images of H&E staining in different groups. (e) KIM-1 protein levels were assessed by western blot analysis. (f) The established inhibitor 4-PBA effectively restrained the protein expression of GRP78, CHOP, and caspase-12 in renal I/R injury. (g) The inhibition of ER stress by 4-PBA tremendously depressed the caspase-1 activity in mice with renal I/R surgery. (h) The mRNA levels of NLRP3, ASC, and caspase-1 in the I/R group declined after the application of 4-PBA. (i, j) 4-PBA as one specific inhibitor of ER stress remarkably depressed the activation of pyroptosis-related protein markers including NLRP3, ASC, caspase-1, GSDMD-N, IL-1*β*, and caspase-11. (k) The protein levels of apoptotic markers consisting of BAX, Bcl-2, and cleaved caspase-3. Values measured during animal experiments were carried out as mean ± SD, *n* = 3 − 5. ^∗^*P* < 0.05, compared with the Sham group; ^#^*P* < 0.05, relative to the NS+I/R group.

The function of ER stress as a key mediator in renal I/R injury was investigated by the utilization of its established inhibitor, 4-PBA. In Figures 4(a) and 4(b), those results showed that preapplication of 4-PBA prior to establishment of renal I/R model clearly downregulated serum blood Cr and BUN levels. Moreover, H&E staining of the kidneys suggested that I/R-induced kidney tissue damage was evidently ameliorated after 4-PBA treatment (Figures 4(c) and 4(d)), which was again corroborated by the KIM-1 protein levels through detection using western blot analysis (Figure 4(e)). As shown in Figure 4(f), preapplication of 4-PBA prior to renal I/R model clearly prevented the initiation of ER stress, as summarized by the downregulated expression in GRP78, CHOP, and caspase-12 proteins. Interestingly, we further innovatively discovered that ER stress was capable of regulating pyroptosis and apoptosis in animal model of renal I/R injury. As confirmed by the experimental discoveries, the inhibition of ER stress by 4-PBA tremendously depressed the caspase-1 activity in mice with renal I/R surgery (Figure 4(g)). The noteworthy attenuation of elevated mRNA levels of NLRP3, ASC, and caspase-1 induced by I/R surgery was displayed after specific inhibition of ER stress using 4-PBA (Figure 4(h)). The remarkable differences in protein levels of NLRP3, ASC, cleaved caspase-1, GSDMD-N, caspase-11, and mature IL-1*β* between the NS+I/R group and 4-PBA+I/R group again testified that renal I/R-generated pyroptosis could be regulated by ER stress in mice (Figures 4(i) and 4(j)). 4-PBA administration also resulted in a striking diminution in the expression of BAX and cleaved caspase-3 proteins and a pronounced upregulation in protein expression of Bcl-2 in the 4-PBA+I/R group (Figure 4(k)).

### 3.5. Naringenin Effectively Alleviated ER Stress in H/R-Exposed HK-2 Cells

**Figure 5:**
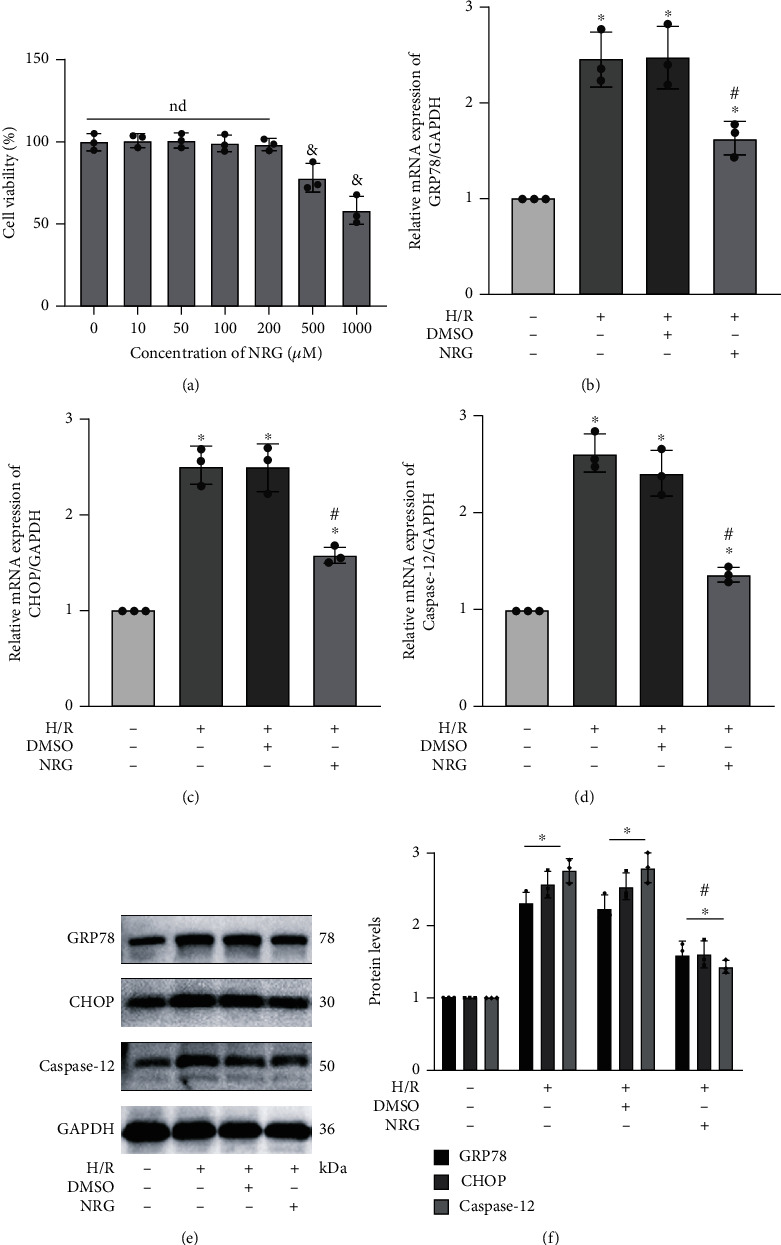
Naringenin effectively alleviated ER stress in H/R-exposed HK-2 cells. (a) CCK-8 assay explored the effects of different concentrations of NAR on cell viability. Concentration at 200 *μ*M was selected in the following experiments. (b–d) The mRNA expression in GRP78, CHOP, and caspase-12 was explored by qRT-PCR. (e, f) Naringenin treatment noticeably alleviated the elevated protein levels of GRP78, CHOP, and caspase-12 in H/R injury. Values measured during cellular experiments were carried out as mean ± SD, *n* = 3 (three independent experiments). ^&^*P* < 0.05, relative to 0 *μ*M group; ^∗^*P* < 0.05, compared with the control group; ^#^*P* < 0.05, versus the DMSO+H/R group; nd: no statistical difference.

In addition, we constructed the H/R model in vitro to further verify the protective effects of naringenin in renal I/R injury. As shown in Figure 5(a), to find out the appropriate concentration that could be used for treating HK-2 cells, we selected CCK-8 assay to explore the effects of different concentrations of NRG on cell viability. The cell activity of HK-2 cells was not significantly affected by NAR at 200 *μ*M, a concentration that was also selected in another study [33]. HK-2 cells were pretreated with 200 *μ*M of NRG dissolved in DMSO and received H/R exposure 24 h later. Quantitative real-time PCR analysis demonstrated that NAR treatment noticeably alleviated the elevated mRNA levels of GRP78, CHOP, and caspase-12 in H/R injury (Figures 5(b)–5(d)). Consistent with mRNA levels, the protein expression of those specific markers of ER stress exhibited the obvious reduction in the NRG+H/R group (Figure 5(e)).

### 3.6. Naringenin Considerably Mitigated H/R-Induced Pyroptosis and Apoptosis In Vitro

**Figure 6:**
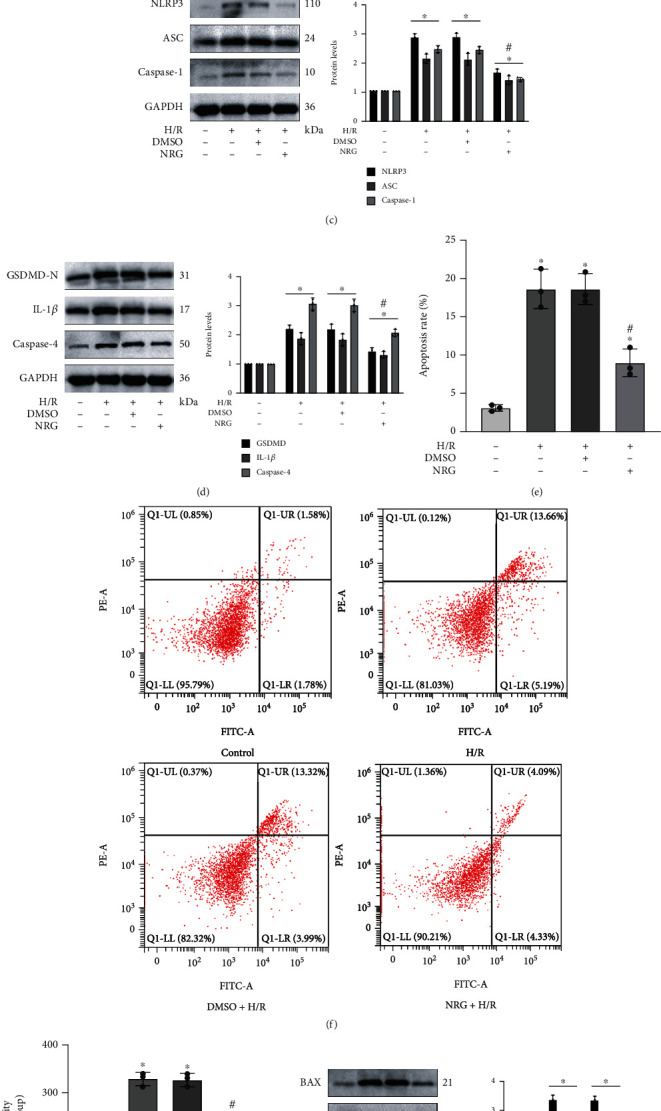
Naringenin considerably mitigated H/R-induced pyroptosis and apoptosis in vitro. (a) The obvious elevation of caspase-1 activity in H/R exposure was remarkably depressed after naringenin application in renal HK-2 cells. (b) The pretreatment of naringenin evidently lessened the mRNA levels of typical pyroptosis-related markers including ASC, NLRP3, and caspase-1 during H/R injury. (c, d) The usage of naringenin markedly decreased the protein levels of representative pyroptosis-related markers such as NLRP3, caspase-1, ASC, GSDMD-N, IL-1*β*, and caspase-4 after H/R exposure. (e, f) The flow cytometry revealed that naringenin administration tremendously mitigated H/R-generated apoptotic HK-2 cells. (g) The caspase-3 activity in various groups. (h) Western blot analysis utilized in protein detection of HK-2 cells was performed to quantify the protein levels of Bcl-2, cleaved caspase-3, and BAX in various group. Values measured during cellular experiments were carried out as mean ± SD, *n* = 3. ^∗^*P* < 0.05, compared with the control group; ^#^*P* < 0.05, versus the DMSO+H/R group.

In Figure 6(a), the obvious elevation of caspase-1 activity in H/R exposure was remarkably depressed after naringenin application in renal HK-2 cells. qRT-PCR revealed that the pretreatment of naringenin evidently lessened the mRNA levels of typical pyroptosis-related markers including ASC, NLRP3, and caspase-1 during H/R injury (Figure 6(b)). Besides, we authenticated that NAR application was capable of attenuating H/R-induced activation of NLRP3 inflammasome in HK-2 cells (Figure 6(c)). Pretreatment of NRG on HK-2 cells prior to the establishment of H/R model remarkably depressed the expression of GSDMD-N, mature IL-1*β*, and caspase-4 proteins, which were crucial markers of pyroptosis (Figure 6(d)). The flow cytometry revealed that NAR administration tremendously mitigated H/R-stimulated apoptotic HK-2 cells (Figures 6(e) and 6(f)). The caspase-3 activity in H/R injury was effectively inhibited by NRG treatment in HK-2 cells (Figure 6(g)). The results of western blot analysis demonstrated that BAX protein levels as well as cleaved caspase-3 protein levels were obviously reduced in the NRG+H/R group versus DMSO+H/R group, whereas Bcl-2 protein levels were upregulated after NRG usage in vitro (Figure 6(h)).

### 3.7. H/R-Induced Pyroptosis and Apoptosis Depended on ER Stress In Vitro

**Figure 7:**
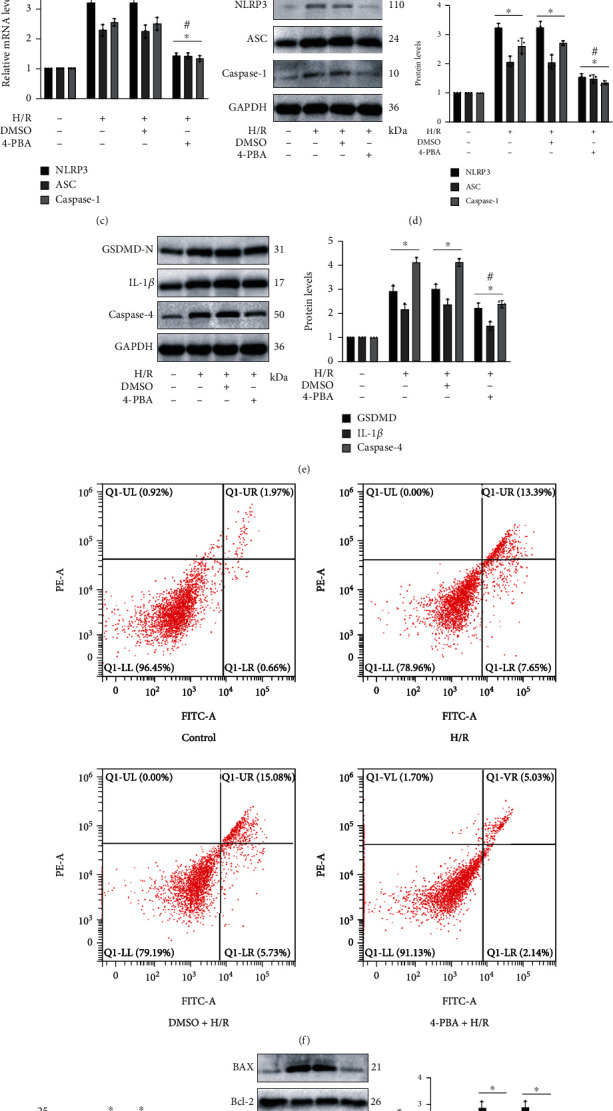
H/R-induced pyroptosis and apoptosis depended on ER stress in vitro. 5 mM of 4-PBA was taken to handle the HK-2 cells 24 h before H/R treatment as described previously. (a) The influence of 4-PBA on GRP78, CHOP, and caspase-12 proteins in H/R injury was verified by western blot analysis. (b) The levels of caspase-1 activity in the four groups. (c) 4-PBA application obviously decreased the relative mRNA levels of NLRP3, caspase-1, and ASC. (d, e) The rising levels of specific protein markers of pyroptosis in H/R injury declined after restraining ER stress by 4-PBA. (f, g) The apoptosis rate in relevant groups was revealed by the flow cytometry. (h) Protein levels of Bcl-2 and cleaved caspase-3 as well as BAX. Values measured during cellular experiments were carried out as mean ± SD, *n* = 3. ^∗^*P* < 0.05, compared with the control group; ^#^*P* < 0.05, versus the DMSO+H/R group.

A concentration at 5 mM of 4-PBA was taken to handle HK-2 cells that were followed by H/R treatment as described previously. Inhibition effect on ER stress of 4-PBA was verified by western blot analysis, as shown by remarkable reduction in expression levels of GRP78, CHOP, and caspase-12 proteins (Figure 7(a)). Notably, 4-PBA application significantly reduced caspase-1 activity and obviously decreased the relative mRNA levels of NLRP3, caspase-1, and ASC in H/R injury (Figures 7(b) and 7(c)). The preusage of 4-PBA also led to the restriction of activated protein markers of pyroptosis during H/R injury (Figures 7(d) and 7(e)). By performing flow cytometry, we could clearly observe that apoptosis rate in HK-2 cells increased considerably during H/R injury, whereas it was greatly reversed after ER stress inhibition through 4-PBA application (Figures 7(f) and 7(g)). The protein levels in different treatment groups confirmed that the administration of 4-PBA effectively restrained the expression of apoptotic markers including BAX and cleaved caspase-3, and the trend of Bcl-2 protein was elevated in the 4-PBA+H/R group (Figure 7(h)).

### 3.8. Naringenin Activated Nrf2/HO-1 Signaling Pathway to Block Endoplasmic Reticulum Stress in HK-2 Cells

**Figure 8:**
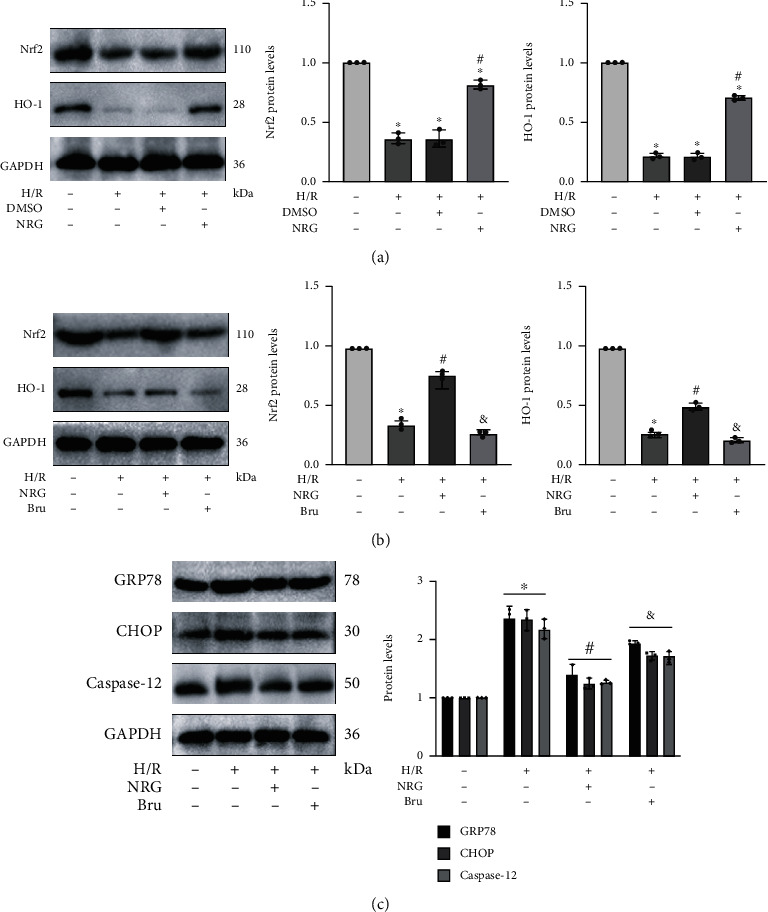
Naringenin activated Nrf2/HO-1 signaling pathway to block endoplasmic reticulum stress in HK-2 cells. 200 *μ*M of NRG was added to the medium 24 h before model construction, and then, they were incubated with or without brusatol (400 nM) 2 h before hypoxia. (a) Naringenin activated Nrf2 and HO-1 after H/R injury. (b) Western blot analysis proved that Nrf2 and HO-1 protein levels were effectively prevented by brusatol (Bru) in HK-2 cells. (c) Inhibiting Nrf2/HO-1 signaling pathway reversed the function of naringenin on restraining ER stress. Values measured during cellular experiments were carried out as mean ± SD, *n* = 3. ^∗^*P* < 0.05, versus the control group; ^#^*P* < 0.05, compared with the H/R group; ^&^*P* < 0.05, relative to the NRG+H/R group.

Next, subject members explored underlying mechanism by which NAR was capable of modulating ER stress. Since NAR could notably alleviate ER stress [34, 35] and Nrf2/HO-1 pathway had been demonstrated to be of great importance in regulating ER stress [36, 37], we examined the expression of relevant proteins after NAR usage in vitro. The protein expressions of Nrf2 and HO-1 were indeed visibly suppressed by H/R treatment, while pretreatment of NRG reactivated Nrf2/HO-1 signaling pathway in H/R injury (Figure 8(a)). We employed brusatol (Bru), an established inhibitor of Nrf2 pathway, to further investigate whether Nrf2/HO-1 signaling pathway exerted effect on NAR-regulated ER stress generated by H/R exposure. The triggering action of NRG on Nrf2/HO-1 signaling pathway was apparently abolished by Bru administration (Figure 8(b)). Interestingly, the abrogating consequence of Bru on Nrf2/HO-1 signaling pathway was accompanied by this striking reversal of NRG's impact on mitigating H/R-generated ER stress in vitro as well (Figure 8(c)).

### 3.9. Schematic Illustration of the Protective Effects of Naringenin on Renal I/R Injury

**Figure 9:**
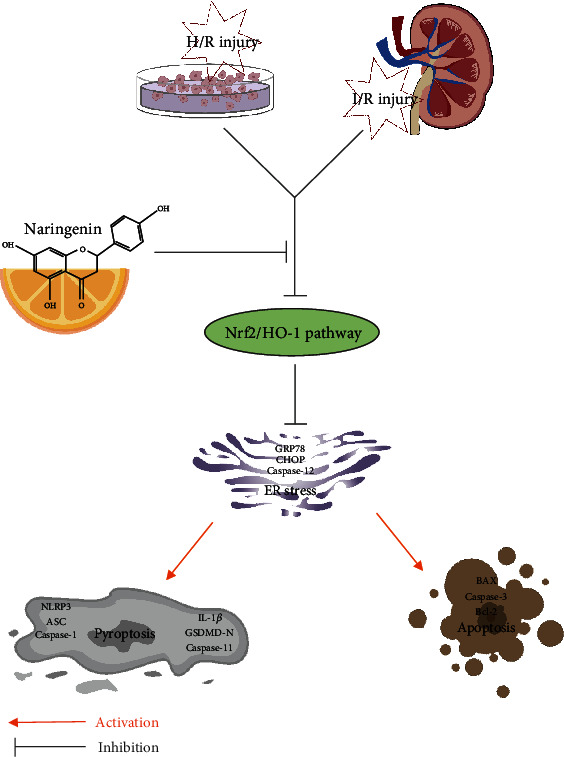
Schematic illustration of the protective effects of naringenin on renal I/R injury.

In vivo and in vitro, naringenin treatment reactivated Nrf2/HO-1 signaling pathway to block ER stress, thus attenuating pyroptosis and apoptosis to exert its protective effects on renal I/R injury (Figure 9).

## 4. Discussion

Renal I/R injury is seen as one complicated and intractable pathological process, and it is usually caused by sepsis, organ transplantation, and renal surgery [5, 7]. What is more serious and unacceptable is that if effective and timely measures are not taken to prevent it, renal I/R injury is prone to gradually develop into AKI, one clinic syndrome with noticeable hospital mortality due to its rapid kidney dysfunction and few satisfactory treatment strategies [1, 38, 39]. Encouragingly, there are also relevant clinical studies, which have demonstrated that some treatment strategies may be able to improve acute kidney injury to some extent by inhibiting pathological events such as ER stress, inflammatory response, and apoptosis in hospital patients. Tang et al. performed clinical trials to confirm that dexmedetomidine could attenuate AKI as well as postischemic myocardial injury, and the mechanisms may be related to the inhibition of ER stress, oxidative stress, and apoptosis [40]. 80 mg/d of atorvastatin may attenuate contrast-induced acute kidney injury by inhibiting apoptosis [41]. Remote ischemic preconditioning displayed significant anti-inflammatory effects [42] and could prevented contrast medium-induced nephropathy [43]. High-dose erythropoietin may be beneficial for some patients with sepsis-AKI possibly through anti-inflammatory effects in macrophage [44]. Naringenin was selected to investigate its role and involved mechanisms in renal I/R injury in our study, mainly because of its good bioavailability, powerful antioxidant, anti-inflammatory and antiapoptotic functions, and wide application prospects. In this research, we smoothly established classical animal model and HK-2 cell model to address the question whether naringenin could attenuate renal I/R injury and the underlying mechanisms behind its protective effects. Primarily, this study innovatively confirmed that NAR significantly improved tissue damage and renal function by inhibiting ER stress, pyroptosis and apoptosis induced by renal I/R injury both in vivo and in vitro. Interestingly, the application of 4-PBA as one characteristic inhibitor of ER stress clearly authenticated that renal I/R-generated pyroptosis and apoptosis were at least partially dependent on ER stress in animal and cell models. Furthermore, the reason for effective mitigation on renal I/R-induced ER stress by naringenin was found to be NAR's power in activating the antioxidant Nrf2/HO-1 signaling pathway. Taken together, the in vivo and in vitro experiments suggested that naringenin activated Nrf2/HO-1 pathway that was notably restrained during the process of renal I/R injury to relieve ER stress, thereby alleviating pyroptosis and apoptosis to protect the kidney against I/R injury.

ER stress, pyroptosis, and apoptosis are regarded as markedly crucial and interrelated molecular events in pathogenesis of renal I/R injury and have received increasing attention in recent years [3, 8, 45]. Endoplasmic reticulum (ER), one indispensable intracellular organelle, is capable of maintaining protein homeostasis such as polypeptide folding, protein modification and degradation, calcium storage, and lipid synthesis [46]. However, unfolded protein response (UPR) will be triggered by pathological conditions including severe hypoxia, persistent calcium imbalance, and destroying ER homeostasis. The whole abnormal intracellular biological activities and stress stimuli in ER eventually induce what is known as endoplasmic reticulum stress [47]. GRP78 normally functions by binding to three major transmembrane protein sensors (ATF6, IRE1, and PERK) of ER stress in the endoplasmic reticulum lumen [48]. GRP78 is able to bind to unfolded proteins and lately could fold or degrade them through ER-related protein degradation pathways. Elevated expression of GRP78 is considered to be one significant marker of ER stress [49]. The degree and duration of pathogenic stimuli could largely determine the ultimate fate of cells, which means that sustained and extreme stress inevitably activates apoptotic UPR pathways to cause apoptosis [50]. Meantime, acting as one common element after the activation of 3 sensors of ER stress mentioned above, CHOP is treated as one crucial regulator of cellular apoptosis induced by ER stress because it could lend assistance in caspase activation, restraining the expression of antiapoptotic Bcl-2, leading to apoptotic cell death [51, 52]. Caspase-12 is a key molecule in the endoplasmic reticulum-specific apoptotic signaling pathway and is not associated with nonendoplasmic reticulum stress-mediated apoptosis. Caspase-12 is localized in the endoplasmic reticulum membrane and undergoes significant activation upon sustained ER stress, which ultimately induces the onset of apoptosis [53]. In I/R injury, related articles confirmed that inhibition of ER stress could effectively suppress apoptosis to alleviate I/R injury [54–56]. Consistent with these studies, our research demonstrated that the pretreatment of 4-PBA as one specific inhibitor of ER stress remarkably attenuated renal I/R-induced apoptosis, as indicated by flow cytometry, depressed expression of cleaved caspase-3, and BAX as well as the recovered levels of Bcl-2 in C57Bl/6 mice and HK-2 cells.

Pyroptosis, one unique form of cell death, is shown to be involved in the occurrence and pathogenesis of AKI along with apoptosis, ferroptosis, and necrosis [57]. Two major pathways are thought to have participated in the occurrence of pyroptosis. The classical signaling pathway is principally regulated through activating caspase-1, while caspase-11 or caspase-4 in human plays an important role inside the nonclassical pathway [8]. Interestingly, pyroptosis is characterized by the noteworthy involvement of inflammatory response especially the activation of NLRP3 inflammasome that was composed of procaspase-1, ASC, and NLRP3. Inactive NLRP3 that was localized in the ER and its adaptor protein ASC induced disruption of mitochondrial homeostasis and initiated pyroptosis upon the process of NLRP3 activation [58]. In addition, ER stress occurring in the early stage is able to induce renal inflammation and activate NF-*κ*B signaling pathway, which incidentally exacerbates the occurrence of pyroptosis [59, 60]. During the procedures of pyroptosis initiation, NLRP3 inflammasome naturally activates caspase-1, which not only drives IL-1*β* precursor into forming mature proinflammatory cytokine IL-1*β* but also cleaves gasdermin D and releases its N-terminal domain, one specific marker binding to membrane lipids, causing pyroptosis [61, 62]. In our investigation, the obvious participation of pyroptosis in renal I/R injury was verified by increased expressions of mentioned markers including caspase-1, ASC, NLRP3, GSDMD-N, caspase-11, caspase-4, and IL-1*β* in animal and cell models. These findings were in agreement with our published articles [25, 29]. Moreover, we innovatively revealed that inhibition of ER stress in renal I/R injury not only considerably relieved renal tissue damage but also successfully mitigated the outbreak of pyroptosis, as evidenced by distinguished alleviation of pyroptosis-related markers mentioned above in the 4-PBA+I/R (or H/R) group compared with the I/R (or H/R) group. Taken together, these data demonstrated that the inhibitory effect of NRG on pyroptosis and apoptosis was at least partially dependent on the alleviation of endoplasmic reticulum stress. The phenomenon that suppression of ER stress was capable of alleviating pyroptosis was also testified in other disease models [63–65].

Naringenin, one polyphenolic constituent existing in dietary citrus fruits, has attracted notable attention from researchers in various fields because of its powerful pharmacological activities and promising therapeutic prospects [32]. Naringenin was proved to regulate inflammatory response, oxidative stress, ER stress, and apoptosis as well as possessing protective effects against common clinical disorders including diabetes, carcinomas, cardiovascular, and neurodegenerative [17, 31]. Fortunately, more than ten clinical trials have been conducted to explore its specific outcome in hospital problems such as cardiovascular diseases, endothelial function, weight control, and HCV infection [14]. Nevertheless, there are no clinical trials of naringenin in kidney diseases, and what is more frustrating is that the role of naringenin in renal I/R injury has not even been fully clarified in basic studies in animal and cell models. During renal I/R injury, naringenin application mitigated the levels of kidney tissue damage, Cr, BUN, and KIM-1 as well as restraining the elevation of relevant markers including CHOP, GRP78, BAX, caspase-12, cleaved caspase-3, caspase-1, ASC, NLRP3, GSDMD-N, caspase-11, caspase-4, and IL-1*β*, indicating that naringenin administration by gavage noticeably attenuated renal tissue damage and improved impaired kidney function in renal I/R injury by preventing ER stress, pyroptosis, and apoptosis, which were proved again in HK-2 cell model.

Nrf2, one nuclear transcription factor, serves as a key regulator in mediating intracellular antioxidant defense system [66]. Although Nrf2 is bound to Keap1 in the cytoplasm and in an inactivated state, Nrf2 separates from its inhibitor protein Keap1 in response to pathological stimuli including oxidative stress and is transferred to the nucleus to activate the downstream genes to exert its antioxidant effects by forming a heterodimer with Maf protein [67]. Among these downstream targets, HO-1 is extremely susceptible to be activated by Nrf2 because it owns the highest number of AREs in the promoter, which is exactly the structure that Nrf2 needs to bind to activate antioxidant proteins [68]. Numerous studies pointed out that Nrf2/HO-1 activation was not only involved in various diseases, development, and oxidative stress response but also effective in relieving renal I/R injury [69]. In addition, with more and more in-depth studies, relevant articles revealed that apart from regulating oxidative stress, Nrf2/HO-1 pathway occupied an important position in regulating apoptosis, inflammation, and endoplasmic reticulum stress [36, 70, 71]. In this research, we observed that the crucial reason for NRG's ability to preserve kidney against I/R injury was its advantages in inhibiting pyroptosis and apoptosis by effectively reducing ER stress. It was frequently demonstrated that Nrf2/HO-1 signaling pathway took a momentous part in moderating ER stress [36, 37, 72], and NRG had been confirmed to have a talent in regulating Nrf2/HO-1 pathway to exert its protective effects [73–75]. The western blot analysis authenticated that decreased protein levels of Nrf2 and HO-1 during renal I/R injury were elevated again after pretreatment of naringenin in animals and HK-2 cells. Moreover, the experiments suggested that NRG could not exert an effect on suppressing H/R-generated ER stress after the addition of brusatol (a well-known Nrf2 inhibitor) in HK-2 cells. In other words, all those figures verified that naringenin blocked ER stress induced by renal I/R injury through activating Nrf2/HO-1 signaling pathway.

## 5. Conclusions

In conclusion, this implemented study innovatively confirmed that naringenin administration was capable of protecting kidney against I/R injury by attenuating pyroptosis and apoptosis via inhibiting ER stress through activating Nrf2/HO-1 signaling pathway. All this evidence could present novel mechanistic insights into the beneficial functions of naringenin, indicating the possibility of a new therapeutic drug for clinical treatment in hospital patients with ischemic kidney disease. However, whether naringenin can regulate other molecular mechanisms to relieve renal I/R injury and the corresponding clinical trials demands to be further explored.

## Figures and Tables

**Table 1:** The primer sequences of human genes used in quantitative real-time PCR analysis.

Primer	Forward primer (5′ ->3′)	Reverse primer (5′ ->3′)	Amplicon size (bp)
GRP78	CACGGTCTTTGACGCCAAG	CCAAATAAGCCTCAGCGGTTT	215
CHOP	GGAAACAGAGTGGTCATTCCC	CTGCTTGAGCCGTTCATTCTC	116
Caspase-12	AACAACCGTAACTGCCAGAGT	CTGCACCGGCTTTTCCACT	118
NLRP3	CGTGAGTCCCATTAAGATGGAGT	CCCGACAGTGGATATAGAACAGA	191
ASC	TGGATGCTCTGTACGGGAAG	CCAGGCTGGTGTGAAACTGAA	110
Caspase-1	TTTCCGCAAGGTTCGATTTTCA	GGCATCTGCGCTCTACCATC	54
GAPDH	ACAACTTTGGTATCGTGGAAGG	GCCATCACGCCACAGTTTC	101

**Table 2:** The primer sequences of mouse genes used in quantitative real-time PCR analysis.

Primer	Forward primer (5′ ->3′)	Reverse primer (5′ ->3′)	Amplicon size (bp)
GRP78	ACTTGGGGACCACCTATTCCT	ATCGCCAATCAGACGCTCC	134
CHOP	CTGGAAGCCTGGTATGAGGAT	CAGGGTCAAGAGTAGTGAAGGT	121
Caspase-12	AGACAGAGTTAATGCAGTTTGCT	TTCACCCCACAGATTCCTTCC	106
NLRP3	ATTACCCGCCCGAGAAAGG	TCGCAGCAAAGATCCACACAG	141
ASC	CTTGTCAGGGGATGAACTCAAAA	GCCATACGACTCCAGATAGTAGC	154
Caspase-1	ACAAGGCACGGGACCTATG	TCCCAGTCAGTCCTGGAAATG	237
GAPDH	AGGTCGGTGTGAACGGATTTG	TGTAGACCATGTAGTTGAGGTCA	123

## Data Availability

Detailed data supporting the findings of this study are included in the manuscript.

## Abbreviations

I/R: Ischemia reperfusion
NRG: Naringenin
H&E: Hematoxylin-eosin
ASC: Apoptosis-associated speck-like protein
AKI: Acute kidney injury
NS: Sterile saline
4-PBA: 4-Phenylbutyric acid
KIM-1: Kidney injury molecule 1
ER stress: Endoplasmic reticulum stress
Cr: Creatinine
GRP78: Glucose-regulated protein, 78 kDa
BUN: Blood urea nitrogen
NLRP3: NLR family pyrin domain containing 3
Nrf2: Nuclear factor- (erythroid-derived 2-) like 2
BAX: BCL2-associated X protein
Keap1: Kelch-like ECH-associated protein 1
GSDMD: Gasdermin D
IRE1: Inositol-requiring protein 1
Bcl-2: B-cell leukemia/lymphoma 2
IL-1*β*: Interleukin-1*β*
HCV: Hepatitis C virus
HO-1: Heme oxygenase (decycling) 1
CCK-8: Cell counting kit 8
ATF6: Activating transcription factor 6
SD: Standard deviation
CHOP: C/EBP-homologous protein
qRT-PCR: Quantitative real-time PCR analysis
Bru: Brusatol
PERK: Protein kinase R- (PKR-) like endoplasmic reticulum kinase
H/R: Hypoxia/reoxygenation
ARE: Antioxidant response element
HPLC: High-performance liquid chromatography
Maf: Muscle aponeurosis fibromatous.
